# Factors affecting the visual outcome in acute central serous chorioretinopathy

**DOI:** 10.12669/pjms.331.11664

**Published:** 2017

**Authors:** Qamar Ul Islam, Muhammad Asad Farooq, Mohammad Asim Mehboob

**Affiliations:** 1 Dr. Qamar Ul Islam, FCPS (Ophthalmology), FCPS (VRO). Department of Ophthalmology, PNS Shifa Hospital, Karachi; 2 Dr. Muhammad Asad Farooq, MBBS. Department of Ophthalmology, CMH Multan; 3 Dr. Mohammad Asim Mehboob, MBBS. Department of Ophthalmology, PNS Shifa Hospital, Karachi

**Keywords:** Central Serous Retinopathy, Optical coherence Tomography, Visual acuity

## Abstract

**Objective:**

To determine the visual outcome in patients with acute Central serous chorioretinopathy (CSCR) and to analyze the association of clinical, angiographic and tomographic factors with final visual outcome in Pakistani population.

**Methods:**

This study was conducted at AFIO Rawalpindi and PNS Shifa Naval hospital Karachi from November 2011 to August 2016. Fifty five eyes of 53 patients with acute CSCR were included. All patients underwent a detailed ophthalmic examination including SD OCT imaging at baseline, One month and three month and FFA was performed at baseline. Primary outcome measures were measurement of initial and final BCVA and CFT. SPSS 13.0 was used for the analysis of data.

**Results:**

Mean age of study population was 36.66 ± 6.24 years. On OCT mean CFT at baseline was 467.49 ± 144.80 µm in affected eye, whereas mean CFT measurements at final follow up was 244.67 ± 32.99 µm (p <0.01). Presenting mean log MAR BCVA was 0.47 ± 0.25 and final mean log MAR BCVA was 0.18 ± 0.14 (p <0.01). Baseline BCVA showed statistically significant association with final BCVA (p=0.03).

**Conclusion:**

Presenting VA of 6/12 or better is associated with favorable visual outcome in patients with acute CSCR.

## INTRODUCTION

Central serous chorioretinopathy (CSCR), first described by Von Graefe in 1866 is an idiopathic disorder of the outer blood retinal barrier, characterized by a localized serous detachment of retina and/or retinal pigment epithelium (RPE) in macular area.^1^,^2^ It is typically a unilateral disorder predominantly affecting males in their 3^rd^ and 4^th^ decades of life.^2^ The pathogenesis of the disease is not clearly understood, but the postulated pathophysiology include stasis, ischemia, and/or inflammation of inner choroid that leads to hyper permeable choroidal vasculature, secondary RPE changes and neurosensory retinal detachment.^3^,^4^ Known systemic risk factors associated with CSCR include type A personality, stress, hypertension, smoking, exogenous steroid use, pregnancy, acid peptic disease, Helicobacter pylori infection, obstructive sleep apnea, alcohol use, use of sympathomimetic agents, use ofphosphodiesterase-5 inhibitors (Sildenafil) and collagen vascular disease (SLE, Sarcoidosis).^2^,^5^,^6^

CSCR has two main forms: acute and chronic. Acute CSCR is characterized by sudden painless central visual deterioration and usually have favorable prognosis with spontaneous recovery in 80%-90% of cases within 2-3 months.^4^ Whereas, chronic CSCR is labeled when sub retinal fluid (SRF) persists for more than three months and has far worse visual prognosis as compared to acute CSCR.^4^ Optical coherence tomography (OCT) and fundus fluorescein angiography (FFA) are the mainstay investigations that are helpful in confirmation of clinical diagnosis, quantification of serous accumulation, documentation of pattern and site of leakage and monitoring of recovery.

Visual prognosis in acute CSCR is usually dependent on presenting visual acuity (VA), duration of symptoms, OCT and FFA features and presence of risk factors. Patients with initial VA of less than 6/9 recover two to three Snellen lines during the course of recovery, whereas patients with presenting VA of 6/6 usually maintain that vision.^5^ Nair et al. in their study reported that in acute CSCR, poorer VA showed statistically significant association with greater dimension of SRF and thinning of outer nuclear layer at fovea.^7^ Aggio et al. found that initial VA may be a reliable predictor of the visual outcome in CSCR.^8^ The objective of this study was to evaluate the visual outcome in patients with acute CSCR and to determine the association between various factors and final visual outcome in Pakistani population.

## METHODS

This prospective case series was conducted at Armed Forces Institute of Ophthalmology (AFIO) Rawalpindi and PNS Shifa Naval Hospital Karachi from November 2011 to August 2016. After approval of hospital ethical review committee, consecutive patients with acute CSCR who presented in Eye OPD during study period were included and informed written consent was obtained from each patient. Acute CSCR was defined as cases with typical symptoms (decreased vision, metamorphopsia) for less than three months duration and diagnosis confirmed on the basis of clinical examination and OCT/FFA findings by an experienced ophthalmologist. Patients with recurrent and chronic CSCR, intraocular inflammation, intraocular surgery, glaucoma or any other macular pathology were excluded. All patients underwent a detailed ophthalmic examination including measurement of Snellen best corrected visual acuity (BCVA), slit lamp biomicroscopy with 90 D lens and spectral domain OCT imaging using Topcon 3 D-1000 Mark-II OCT machine at baseline, one month and three month. The height of the SRF labeled as central foveal thickness (CFT) was measured as the greatest distance from RPE to the border of the detached neurosensory retina, which was measured automatically within one mm central diameter of the fovea. FFA was performed at baseline in patients with no contraindications to angiography with Topcon TRC -50EX machine and pattern and site of leakage was documented. All the patients were managed conservatively with control of modifiable risk factors and topical 0.1% Nepafenac (Nevanac, Alcon Lab) three times a day for at least three months. Detailed history with documentation of known risk factors, demographic profile, clinical findings, laboratory investigations (serum Cortisol and serum testosterone levels), OCT and FFA findings were endorsed on a pre devised proforma in each patient. For statistical analysis Snellen BCVA was converted to Log MAR values using online VA converter. Primary outcome measures were measurement of initial and final BCVA and CFT.

SPSS 13.0 was used for the analysis of data. Results were expressed as mean ± Standard deviation (SD)/median and Interquartile range (IQR) for quantitative variables (age, duration of symptoms, OCT values, and hormonal levels) and frequencies and percentages for qualitative variables (gender, laterality, risk factors, and angiographic features, visual outcome). Association of various variables with final visual outcome was analyzed using Chi square test/Fischer exact test. Correlation between various variables was analyzed using Pearson correlation. Paired sample ‘t’ test was used to compare initial and final BCVA and CFT. A p < 0.05 was considered significant.

## RESULTS

Fifty five eyes of 53 patients fulfilling the inclusion criteria were eligible for final analysis. Mean age of study population was 36.66 ± 6.24 years (range: 24 -54 years) with 64% of patients in their 4^th^ decade of life. Clinical profile of study population is given in Table-I. Median duration of visual involvement at the time of presentation was 12 days (IQR: 6.50-30 days). Thirty one (58.49%) patients presented within two weeks of onset of disease. Known systemic risk factors for CSCR were present in 45 (84.71%) patients. On OCT mean CFT at baseline was 467.49 ± 144.80 µm (range: 208-891 µm) in affected eye as compared to 233.41 ± 21.24 µm (range: 184- 288 µm) in normal eye (p < 0.05), whereas mean CFT measurements at final follow up was 244.67 ± 32.99 µm which was statistically significant as compared to baseline values (p <0.01) (Table-II). There was also a significant positive correlation of final BCVA with final OCT measurements of affected eye (Pearson r = 0.517, p <0.01) and OCT values of other eye (r = 0.292, p =0.037). Whereas, correlation between final BCVA and baseline OCT values was not significant (r = 0.212, p = 0.12).

**Table-I:** Clinical characteristics of the study population.

Characteristic	No (%)
*Gender (n= 53)*	
Male	47 (88.67%)
Female	6 (11.32%)
*Eye Involved (n= 53)*	
Right	21 (39.62%)
Left	30 (56.60%)
Bilateral	2 (3.77%)
*Number of Risk Factors (n= 53)*	
0	8 (15.09%)
1	22 (41.50%)
2	15 (28.30%)
3 or more	8 (15.09%)
*FFA (n= 41)*	
Ink Blot pattern	22 (53.65%)
Smoke Stack pattern	6 (14.63%)
Non-specific pattern	13 (31.70%)

**Table-II:** Initial and Final CFT and Visual Acuity profile.

Variable	Baseline (n=55)	95% CI	Final (n=55)	95% CI	p Value
*CFT (µm) Mean ± SD*	467.49 ± 144.80	429.22 – 505.76	244.67 ± 32.99	235.95 – 253.39	< 0.01
*BCVA Log MAR*	0.47± 0.25	0.41 - 0.54	0.18 ± 0.14	0.14 – 0.22	< 0.01

Presenting mean log MAR BCVA was 0.47 ± 0.25 (range: 0.10- 1.0) and final mean log MAR BCVA was 0.18 ± 0.14 (range: 0.0 – 0.60) (p <0.01). There was strong positive correlation between initial and final BCVA, that was statistically significant (r = 0.632, *p* = 0.000). Association between final BCVA and variables like duration of symptoms, baseline CFT, number of risk factors, FFA pattern and baseline BCVA was analyzed. Out of these variables only the baseline BCVA showed statistically significant association with final BCVA (p=0.03) (Table-III).

**Table-III:** Association of Study Variables with Final Visual Outcome.

Variable	Final BCVA n (%)		p value*

	(6/12 or better)	(6/15 or worse)	
Duration of Symptoms (n=53)			
< 2 Weeks	29 (54.71%)	2 (3.77%)	0.354
> 2 weeks	18 (33.96%)	4 (7.54%)	
*Baseline CFT (n=55)*			
>400 µm	29 (52.72%)	4 (7.27%)	1.00
<400 µm	20 (36.36%)	2 (3.63%)	
*Number of Risk factors (n=53)*			
0	7 (13.20%)	1 (1.88%)	0.826
1	19 (35.84%)	3 (5.66%)	
2	13 (24.52%)	2 (3.77%)	
3 or more	8 (15.09%)	0 (0.00%)	
*FFA Pattern (n=41)*			
Ink Blot	21 (51.21%)	1 (2.43%)	0.670
Smoke Stake	5 (12.19%)	1 (2.43%)	
Non Specific	11 (26.82%)	2 (4.87%)	
*Baseline BCVA (n=55)*			
6/12 or better	24 (43.63%)	0 (0.00%)	0.030
6/15 or worse	25 (45.45%)	6 (10.90%)	

* Chi Square/Fischer exact test.

## DISCUSSION

Due to usually self-limiting nature of acute CSCR with good visual outcome, conservative approach with observation and addressing the modifiable risk factors remains the mainstay of management in newly diagnosed cases of less than three month duration.^6^,^9^ Acute CSCR usually a unilateral disease affecting young males who presented with sudden onset visual deterioration along tt may or may not be associated with metamorphopsia. Mean age of our study population was 36.66 ± 6.24 years with 88.67% of patients were male and 96.22% had unilateral involvement. Studies on Pakistani population with CSCR have reported mean age range from 39.09 to 40 years, male preponderance (81.3%-90.19%) and unilateral involvement in 65.5% cases.^10^,^11^

Various other studies from all over the world have reported mean age range from 40-44.7 years, male predominance between 74.60-90% and unilateral involvement in 58-91.63% in patients with CSCR.^1^,^7^,^12^-^14^ Most common angiographic patterns in our study were ink blot pattern (53.65% eyes) and smoke stack pattern (14.63% eyes). Jamil et al.^10^ and Khalil et al.^11^ reported ink blot appearance in 74.4% and 52.94% of Pakistani population with CSCR. SD-OCT provide quick, non-invasive and reproducible high-definition images of the retinal layers and is the primary imaging modality for the diagnosis and follow-up of CSCR. On OCT, acute CSCR appears as an elevation of the full-thickness neurosensory retina from the highly reflective RPE–choriocapillaris complex separated by an optically empty zone with or without RPE detachments and significantly increased choroidal thickness in patients.^15^,^16^

In our study mean CFT measurement at baseline was 467.49 µm in affected eye which was significantly higher as compared to CFT values in normal eyes (233.41 µm) and at final follow up in affected eyes (244.67 µm). Kim et al in a case series of 63 eyes with first episode unilateral acute CSCR found that CFT at baseline was 528.8 μm and at final follow up was 233.9 μm (p<0.001).^12^ Jamil et al in their study of patients with CSCR treated with intravitreal Bevacizumab injections reported a statistically significant difference between the baseline median OCT thickness (557 µm) and 6 month follow up values (286 µm).^10^ In another series of cases of CSCR with spontaneous resolution mean baseline OCT macular thickness was 473 µm and that improved to 269 µm at 3 months follow up.^17^

Visual prognosis in acute CSCR is usually favorable with complete recovery in most of the cases after first episode. However, presenting VA, duration of symptoms, OCT and FFA features and presence of risk factors may affect the visual outcome. In our study, presenting mean log MAR BCVA was 0.47 and final mean log MAR BCVA was 0.18 (p <0.01) with a strong positive correlation between initial and final BCVA. Promising visual results were reported in various international studies with baseline log MAR BCVA ranging from 0.21 -0.40 that had improved to final Log MAR BCVA in the range of 0.00-0.14.^8^-^12^,^13^,^17^ Wong et al. found that acute CSCR that resolved spontaneously or following treatment had a good long term prognosis for visual functions.^18^ In our study, factors like duration of symptoms, baseline CFT, number of risk factors, angiographic features showed no significant association with final visual outcome. However, presenting VA of 6/12 or better was found to be significantly associated with better final visual outcome. In a study by Aggio et al., statistically significant correlation was observed between baseline BCVA as well as duration of symptoms and final BCVA, however, angiographic features were not significantly correlated with visual outcome.^8^ Maalej et al. found that factors associated with poor functional outcome in CSCR were poor initial VA, greater SRF height, and the number of PED’s.^19^ Loo et al. reported that factors associated with reduced VA during long term follow up of patients with idiopathic CSCR included persistent RPE detachment and/or SRF, recurrences and sub macular choroidal neo vascularization.^20^ SD OCT provides detailed insight into the foveal integrity of inner segment/outer segment (IS/OS) and external limiting membrane (ELM) along with other features of CSCR including hyper reflective dots and RPE changes that were found to have direct correlation with final visual outcome. Results of study by Yalcinbayir et al. showed that IS/OS line disruption and loss of integrity of ELM was strongly associated with low visual outcome.^21^ Moreover, no correlation was found between BCVA and overall measurements of CFT, sub foveal choroidal thickness and foveal ONL.^21^ Kim et al. reported a statistically significant difference between the mean BCVA of eyes with visible IS/OS and that of eyes with invisible IS/OS in patients with acute CSCR.^12^ Nair et al concluded that in the acute phase of CSCR, poorer VA showed statistically significant association with greater dimensions of SRF and thinning of the ONL at the fovea, whereas, in resolved CSCR, poorer VA was associated with a persistently thinner ONL, shorter photoreceptor lengths, and IS/OS junction atrophy.^7^ In our study, all patients were managed conservatively with control of modifiable risk factors and topical 0.1% Nepafenac for at least three months. Alkin et al in another study observed complete resolution of macular SRF in 82.3% eyes that were treated with topical Nepafenac as compared to 42.8% eyes with no treatment (p=0.02).^22^

Limitations of this study were relatively shorter follow up that might elude the long term interpretation of functional and anatomical outcomes. Moreover, in depth study of OCT characteristics of study population such as IS/OS integrity, RPE detachments and choroidal thickness was not done that have been proven to be the important factors with regards to anatomical and functional outcomes.

## CONCLUSION

Acute CSCR is usually a self-limiting disorder that usually resolves within three months without any aggressive treatment with good visual outcome. Presenting VA of 6/12 or better is associated with favorable visual outcome in patients with acute CSCR.
